# Disease pathology signatures in a mouse model of Mucopolysaccharidosis type IIIB

**DOI:** 10.1038/s41598-023-42431-4

**Published:** 2023-10-04

**Authors:** Ralitsa Petrova, Abhijeet R. Patil, Vivian Trinh, Kathryn E. McElroy, Minoti Bhakta, Jason Tien, David S. Wilson, Liling Warren, Jennifer R. Stratton

**Affiliations:** 1 Biologics Discovery Science, Teva Pharmaceutical Industries Ltd, Redwood City, CA USA; 2 Genomics and Computational Biology, Teva Pharmaceutical Industries Ltd, West Chester, PA USA

**Keywords:** Diseases of the nervous system, Molecular neuroscience, Mechanisms of disease, Lysosomes, Neurodevelopmental disorders

## Abstract

Mucopolysaccharidosis type IIIB (MPS IIIB) is a rare and devastating childhood-onset lysosomal storage disease caused by complete loss of function of the lysosomal hydrolase α-*N*-acetylglucosaminidase. The lack of functional enzyme in MPS IIIB patients leads to the progressive accumulation of heparan sulfate throughout the body and triggers a cascade of neuroinflammatory and other biochemical processes ultimately resulting in severe mental impairment and early death in adolescence or young adulthood. The low prevalence and severity of the disease has necessitated the use of animal models to improve our knowledge of the pathophysiology and for the development of therapeutic treatments. In this study, we took a systematic approach to characterizing a classical mouse model of MPS IIIB. Using a series of histological, biochemical, proteomic and behavioral assays, we tested MPS IIIB mice at two stages: during the pre-symptomatic and early symptomatic phases of disease development, in order to validate previously described phenotypes, explore new mechanisms of disease pathology and uncover biomarkers for MPS IIIB. Along with previous findings, this study helps provide a deeper understanding of the pathology landscape of this rare disease with high unmet medical need and serves as an important resource to the scientific community.

## Introduction

Lysosomal storage diseases (LSD) are rare heritable monogenic disorders caused by recessive loss-of-function mutations in lysosomal catabolism genes^1,2^. Despite being genetically heterogeneous, LSDs most often present as pediatric neurodegenerative diseases with developmental delay and defects in multiple organ systems. The diagnosis of LSDs is often delayed due to the variability in clinical presentation, especially in milder cases with longer survival and more common symptoms^1,2^. A hallmark of all LSDs, and a key factor in diagnosis, is the accumulation of substrates resulting from the absence of functional lysosomal enzymes normally responsible for their degradation. One class of LSDs typified by a disruption in the degradation process and subsequent storage of mucopolysaccharides/glycosaminoglycans (GAGs) are the mucopolysaccharidoses (MPS). MPS etiology stems from deficiencies in 11 different lysosomal enzymes, which give rise to seven distinct clinical types (MPS I-VII, IX) and additional subtypes (MPS I H/S, MPS III A-D, MPS IV A/B) of MPS. MPS global incidence and prevalence rate vary geographically with type III being one of the most frequently occurring types^3,4^.

MPS III, also known as Sanfilippo syndrome after the medic who first reported on the disease^5^, is caused by loss-of-function in one of four lysosomal enzymes responsible for heparan sulfate (HS) degradation^3^. MPS III subtypes A and B are the most prevalent subtypes of Sanfilippo syndrome making up to 90% of MPS III cases^6^. MPS IIIB, the focus of this study, results from the inactivation of the gene encoding the lysosomal enzyme α-*N*-acetylglucosaminidase (NAGLU)^7,8^, which is responsible for hydrolysing the α1→4 linkage between *N*-acetylglucosamine and the neighboring uronic acid in the latter stages of HS degradation. Over 100 deletions, insertions, missense, nonsense and splice-site mutations in the *NAGLU* coding region have been reported^8–20^, all leading to HS accumulation in tissues and varying degrees of disease severity. In MPS III patients, HS accumulates in lysosomes or is excreted in biofluids when not degraded, allowing diagnosis based on the increased concentration of HS in patient urine^21^. The variability in NAGLU-inactivating mutations and their low allelic frequency, however, have contributed to a rather wide spectrum of clinical phenotypes and severity in pathology^18,22,23^. Currently there are no approved treatments for MPS IIIB, although several therapeutic approaches are under investigation including small molecule therapeutics for substrate reduction, misfolded enzyme chaperones, recombinant enzyme replacement therapeutics and Adeno-associated virus mediated *NAGLU* gene therapies^24^. Disease progression usually becomes apparent in the first 1–4 years of life when MPS IIIB patients begin exhibiting developmental delays and behavioral problems associated with changes in brain anatomy including cortical atrophy, ventricular enlargement and abnormalities in the white matter^23,25^. These are followed by extreme hyperactive behavior, progressive mental decline, motor deterioration and severe dementia before end of life usually in the second decade^26^.

Despite our detailed knowledge of the genetic and molecular drivers of MPS III, our current understanding of how exactly HS accumulation and lysosomal dysfunction lead to global defects in other cell organelles and cell death remains incomplete. Due to the low frequency of MPS IIIB occurrence, as with most LSDs, animal model availability—from rodents to large stock animal species^27–29^, has been a key factor in studying mechanisms of disease pathogenesis and has enabled translational research towards finding a cure. In this study, we provide a systematic and comprehensive histological, behavioral and proteomic analysis of the most commonly used MPS IIIB mouse model—the *Naglu* knock-out (KO) mouse genetic model^30^. While MPS IIIB pediatric patients exhibit symptoms within the first few years of life, *Naglu* KO mice become symptomatic in adulthood with reported onset of behavioral deficiencies around 4–5 months of age and rapid deterioration after 6 months of age. In order to explore the early mechanisms of disease pathology in this MPS IIIB mouse model, we focused our analyses at the pre-symptomatic and early symptomatic phases of disease development in 2- and 5-month-old mice, respectively. We confirmed some aspects of behavioral deficits detected in patients and *Naglu* KO mice over the years, and observed previously unreported liver enlargement in KO animals—reminiscent of the MPS IIIB pathological phenotype reported in patients. We report considerable storage defects in the brain and liver of *Naglu* KO animals, which were readily detectable at the ultrastructural level by 2 months of age. Our histological analysis on *Naglu* KO brains revealed extensive neuronal degeneration and signs of substantial astrogliosis and microglial damage, consistent with previous reports of neuroinflammation in MPS IIIB. In addition, we provide further evidence for the onset of blood–brain barrier deterioration in *Naglu* KOs at earlier stages than previously reported. Finally, our multiplex proteomic analysis of tissues and biofluids from NAGLU-deficient animals uncovered disease-relevant tissue-specific and global changes across matrices, which could be applicable to the identification of disease biomarkers for MPS IIIB. We propose that our findings, in the context of the published literature, are an important resource toward deepening our understanding of MPS IIIB pathology and provide an important tool for systematically studying LSD disease progression in rodent preclinical models.

## Results

### Gross pathological assessment of NAGLU-deficient mice

**Figure 1:**
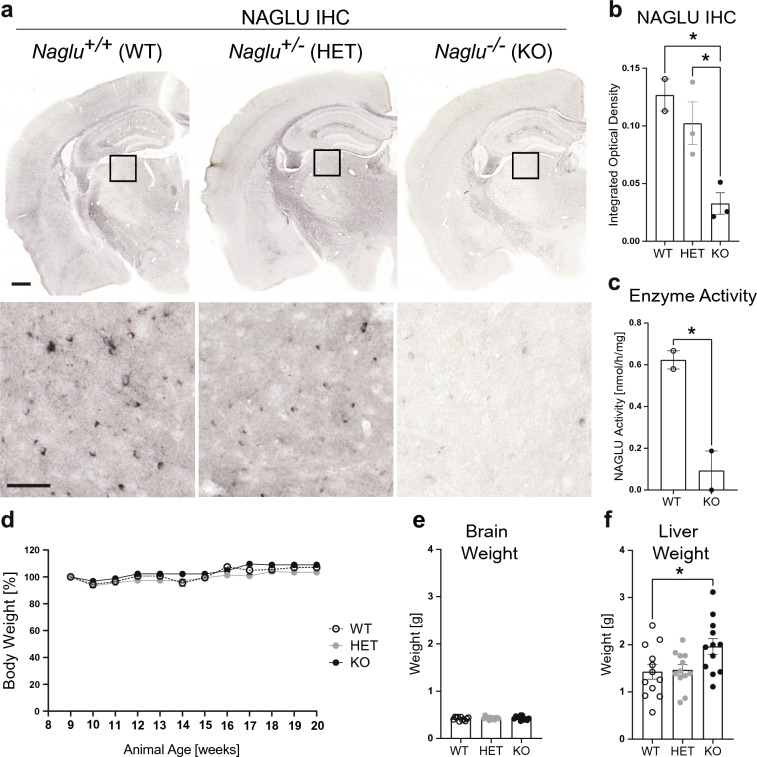
*Naglu* KO mice display hepatomegaly. (**a**) NAGLU immunohistochemistry in 2-month-old WT, HET and KO mouse brains. *Top panel*: coronal sections showing immunohistochemistry (IHC) staining signal decrease in the KO. Scale bar, 500 µm; box indicating location of the Posterior Nucleus of the thalamus. Bottom panel: close-up images from the Posterior Nucleus of the thalamus area in the images above. Scale bar, 50 µm. (**b**) Quantification of NAGLU IHC signal intensity in WT, HET and KO mouse brains at 2-months old (analyzed n = 3 sections/brain, n = 2–3 animals/group). Data shown as mean ± SEM; one-way ANOVA with Tukey’s post-hoc multiple comparisons test; *p < 0.05. (**c**) NAGLU enzyme activity assay in mouse brain tissue demonstrating significantly reduced activity levels in KO mice (analyzed n = 2 brains/group). Data shown as mean ± SEM; unpaired t-test; *p < 0.04. (**d**) *Naglu* KO and littermate control animals (n = 6–12/group) were weighed weekly starting at 2–2.5 months old for 12 weeks. Plot represents changes in weight as percentage (%) of starting weight. Data shown as mean ± SEM; analysis for significance performed using a mixed-effect model (REML) did not show statistical significance (p = 0.0689). (**e**) Total brain weights of *Naglu* KO and control animals (n = 6–12/group) at 2–5 months old. Data shown as mean ± SEM; analysis for significance performed using ordinary one-way ANOVA with Tukey’s post-hoc multiple comparisons test detected no statistical significance. (**f**) Total liver weights of *Naglu* KO and control animals (n = 6–12/group) at 2–5 months old. Data shown as mean ± SEM; one-way ANOVA with Tukey’s post-hoc multiple comparisons test; *p < 0.05.

The targeted disruption of exon 6 of the *Naglu* mouse gene, which represents a genetic region commonly mutated in MPS IIIB patients, renders *Naglutm1Efn/J* a null allele (*Naglu* KO)^30^. Our immunohistochemical (IHC) staining for NAGLU in the brain tissue of 2-month-old mutant and littermate control animals confirmed a dose-dependent reduction in enzyme, which was significant in KO animals (Fig. 1a,b). We detected almost no enzymatic activity in KO brain tissue lysates compared to low levels of activity (~ 0.6 nmol/h/mg) in wild-type littermate controls (Fig. 1c), consistent with brain NAGLU activity being lower than in other organs^30^. Reports on young adult *Naglu* KO animals describe an initially healthy appearance, which deteriorates after 6 months of age with KOs exhibiting urinary bladder and abdominal distention, weight loss, skin ulcerations, bone malformations and decline in motor function, vision, and hearing^30,31^. Consistently, we observed no significant difference in total body weight between groups nor changes in the weight of young *Naglu* KO or control animals over the course of the 12-week assessment period starting at 2-months-old (Fig. 1d). Examination of KO and control brains at 4–5 months of age showed no gross changes in brain weight at this timepoint (Fig. 1e). With hepatomegaly being one of the symptoms in MPS IIIB patients^25^, we next analyzed the livers of 2- to 5-month-old mutant and control animals. Interestingly, we detected a significant increase in liver weight in *Naglu* KO mice compared to controls (Fig. 1f), pointing to a key similarity between the mouse model and MPS IIIB condition in humans. There was no significant effect of age on liver weight when comparing 2 and 5 month mice within each genotype.

### Significant lysosomal storage lesions are present in young *Naglu* KO mice

**Figure 2:**
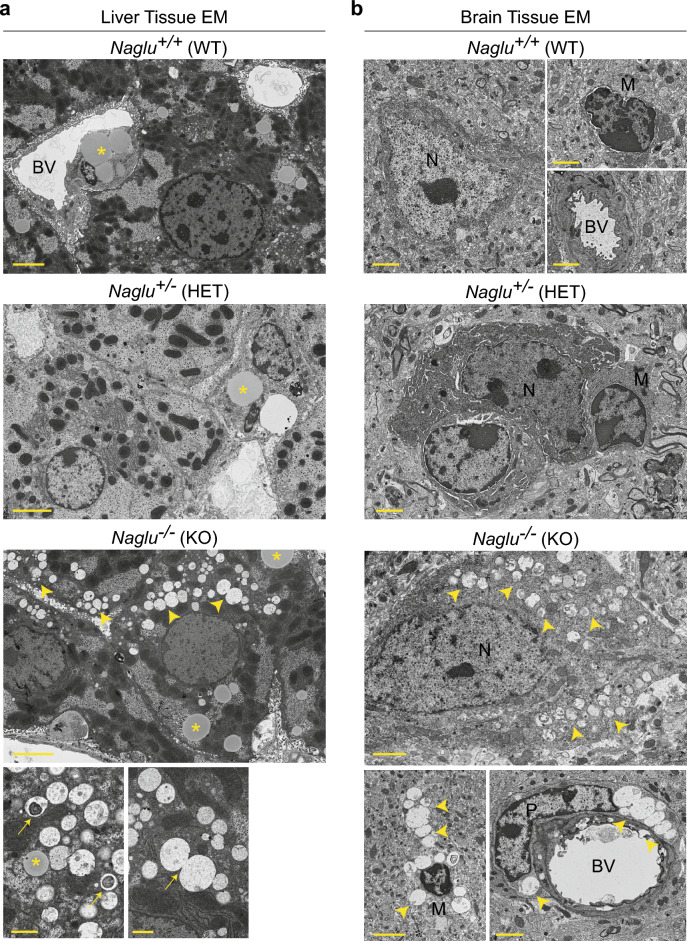
Substrate accumulation is readily detectable in *Naglu* KO mice by 2 months of age. (**a**) Electron micrographs (EM) of liver tissue sections of *Naglu* KO and control animals. Intracytoplasmic glycogen and lipid droplets (*asterisk*) are detectable in all images. Only *Naglu* KO tissues had accumulated many intracytoplasmic vacuoles (*arrowheads*) containing flocculent material and lamellar structures (*arrow, inset bottom left*), which could also be seen coalescing (*arrow, inset bottom right*). BV—blood vessel. Scale bar, 4 µm; insets—1 µm. (**b**) Electron micrographs of brain tissue of *Naglu* KO and control animals showing ultrastructural characteristics of neurons (*N*) and microglia (*M*—presumed). Intracytoplasmic vacuoles (*arrowheads*) are readily detectable in *Naglu* KO brain cells, including neurons, microglia (*inset bottom left*), pericytes (*P*) and perivascular spaces (*inset bottom right*). BV—blood vessel. Scale bar in main and insets, 2 µm.

Next, we analyzed the brains and livers of 2- and 4-month-old *Naglu* KO and control animals for the presence of lysosomal storage lesions that are characteristic of NAGLU loss-of-function and GAG storage in MPS IIIB^30,32–34^. Previous studies have reported the presence of smaller vacuolations at around one month of age in various *Naglu* KO brain regions^30,32^, as well as in liver, spleen, lymph nodes, kidney, lung and skin, with macrophages being the most heavily affected cell type^30,32,35^. Using electron microscopy we detected many vacuoles containing granular flocculent material and amorphous cellular debris in the livers of *Naglu* KO animals at 2 and 4 months of age (Fig. 2a). Vacuolations were present in Kupffer cells as well as in hepatocytes throughout the examined areas. Similarly, we observed heavy vacuolation in the brain motor cortex of *Naglu* KO animals at both stages analyzed. Intracytoplasmic vacuoles were readily detectable in neurons and microglia, as well as in perivascular spaces and blood vessel-adjacent pericytes (Fig. 2b). No vacuolations nor other ultrastructural defects were found in the liver and brain tissues of *Naglu* heterozygous animals at either time point (Fig. 2a,b). Interestingly, we did not detect a drastic deterioration in the degree of vacuolation in *Naglu* KO liver nor brain from 2 to 4 months. In light of this and consistent with previous findings in this model of MPS IIIB^30,32–35^, disease progression likely speeds up more dramatically after the 4-month mark and with the onset of the symptomatic disease stage.

### Widespread neuroinflammation and brain damage in young *Naglu* KO animals

**Figure 3:**
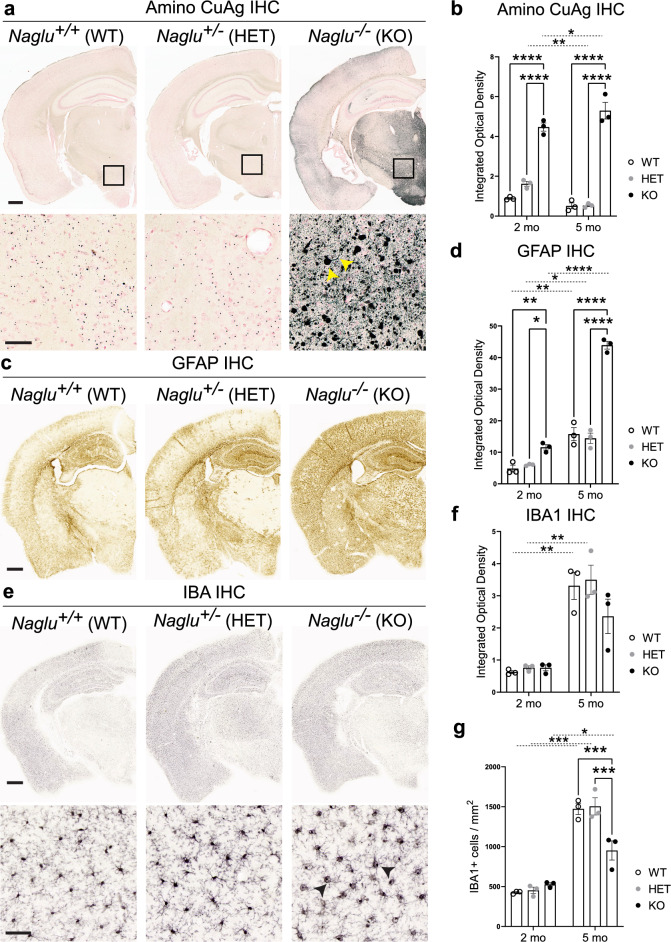
Deletion of *Naglu* leads to early onset of neurodegenerative and inflammatory changes in MPS IIIB mice. (**a**) *Top panel*: Coronal sections from 5-month-old *Naglu* KO and control brains stained with Amino Cupric Silver (Amino CuAg) stain marking degenerating cell bodies, axons and dendrites present in KO animals. Scale bar, 500 µm. *Bottom panel:* Close-ups from the images in the top panel magnifying areas in the hypothalamus revealing heavily stained neuronal bodies and processes (*yellow arrowheads*) in *Naglu* KO brains. Scale bar, 50 µm. (**b**) Quantification of Amino CuAg IHC intensity in coronal brain section images from 2- and 5-month-old *Naglu* KO and control animals (analyzed n = 3 sections/brain in 3 animals/group). Data shown as mean ± SEM; two-way ANOVA with Tukey’s post-hoc multiple comparisons test between genotypes; ****p < 0.0001; two-way ANOVA with Sidak’s post-hoc multiple comparisons test between time points; *p < 0.05; **p < 0.01. (**c**) Coronal sections from 2-month-old *Naglu* KO and control brains stained for Glial Fibrillary Acidic Protein (GFAP) marking astrocytes in the brain. Scale bar, 500 µm. (**d**) Quantification of GFAP IHC staining in coronal brain section images from 2- and 5-month-old *Naglu* KO and control animals (analyzed n = 3 sections/brain in 3 animals/group). Data shown as mean ± SEM; two-way ANOVA with Tukey’s post-hoc multiple comparisons test between genotypes; *p < 0.05; **p < 0.01; ****p < 0.0001; two-way ANOVA with Sidak’s post-hoc multiple comparisons test between time points; *p < 0.05; **p < 0.01; ****p < 0.0001. (**e**) *Top panel*: Coronal sections from 2-month-old *Naglu* KO and control brains stained for the Ionized Calcium Binding Adaptor molecule 1 (IBA1) antigen marking microglial cells in the brain. Scale bar, 500 µm. *Bottom panel:* Close-ups from the images in the top panel magnifying areas in the cortex revealing changes in microglial morphology (*arrowheads*) in *Naglu* KO brains. Scale bar, 50 µm. (**f**,**g**) Quantification of IBA1 IHC in coronal brain section images from 2- and 5-month-old *Naglu* KO and control animals (analyzed n = 3 sections/brain in 3 animals/group), measuring IBA1 intensity (**f**) and number of antigen-positive cells (**g**). Data shown as mean ± SEM; two-way ANOVA with Tukey’s post-hoc multiple comparisons test between genotypes; ***p < 0.001; two-way ANOVA with Sidak’s post-hoc multiple comparisons test between time points; *p < 0.05; **p < 0.01; ***p < 0.0005.

Given the severe neurological deficiencies associated with Sanfilippo syndrome IIIB^26^, we focused our histopathological analyses on the brain. The presence of neuroinflammation in 3 month or older *Naglu* KO mice, microgliosis in particular, has been previously well characterized^34–37^. In order to assess the extent of neuronal damage resulting from GAG accumulation in *Naglu* KO neurons (Fig. 2b), we performed Amino Cupric Silver Stain (Amino CuAg) on brain sections from 2- and 5-month-old mutant and control animals (Fig. 3a,b). Unlike staining for apoptotic or other cell death markers, which label only the cell body, with Amino CuAg staining silver ions bind to denatured proteins in degenerating neurons allowing for detection of irreversible disintegration and structural damage in neuronal axons, dendrites and terminals^38^. We observed dark tint-labelled degenerating neuronal structures in the cortex and subcortical areas of 2- and 5-month-old *Naglu* KO mice, suggesting significant irreversible neurodegeneration throughout the brains of young NAGLU-deficient animals. The severity of neuronal damage was most notable in the thalamic and hypothalamic regions of *Naglu* KO brains (Fig. 3a).

Previous reports have documented the presence of reactive astrocytes, a component of neuroinflammation, throughout the brains of *Naglu* KO mice^34,39^. Our GFAP IHC analysis in 2- and 5-month-old mutant and control brains revealed widespread upregulation in the expression of this glial marker in *Naglu* KOs (Fig. 3c,d). We observed non-overlapping GFAP+ reactive astrocytes with hypertrophic cell bodies and processes across multiple brain areas of *Naglu* KO mice (Fig. 3c), indicative of moderate reactive gliosis^40^. GFAP expression indicative of astrogliosis levels became more exacerbated by 5 months of age in *Naglu* null mutants (Fig. 3d).

Upregulation in microglial inflammatory markers such as CD68 and CD11b among others has been reported in *Naglu* KO mouse brains as early as 3 months of age^34–37^. This is consistent with the extensive buildup of GAG-containing vacuoles detectable at earlier stages in *Naglu* KO microglia (Fig. 2b)^32,33,35^. IHC staining for the pan-microglial marker IBA1 in 2- and 5-month-old mutant and control animal brain sections showed no significant changes in global IBA1 expression intensity or pattern between genotypes or over time (Fig. 3e,f). However, we observed dramatic changes in *Naglu* KO microglial morphology, which appeared atrophied, with swollen cell bodies (Fig. 3e), likely due to the distention of the lysosomal compartment with GAG accumulation^34,36^. Cell count analysis of IBA1 + cells in mutant and control brains also revealed a significant decrease in cell number concomitant with loss of NAGLU function by 5 months of age (Fig. 3g), pointing to deficiencies in the *Naglu* KO microglial population.

### Increased blood–brain barrier permeability in young *Naglu* KO mice

**Figure 4:**
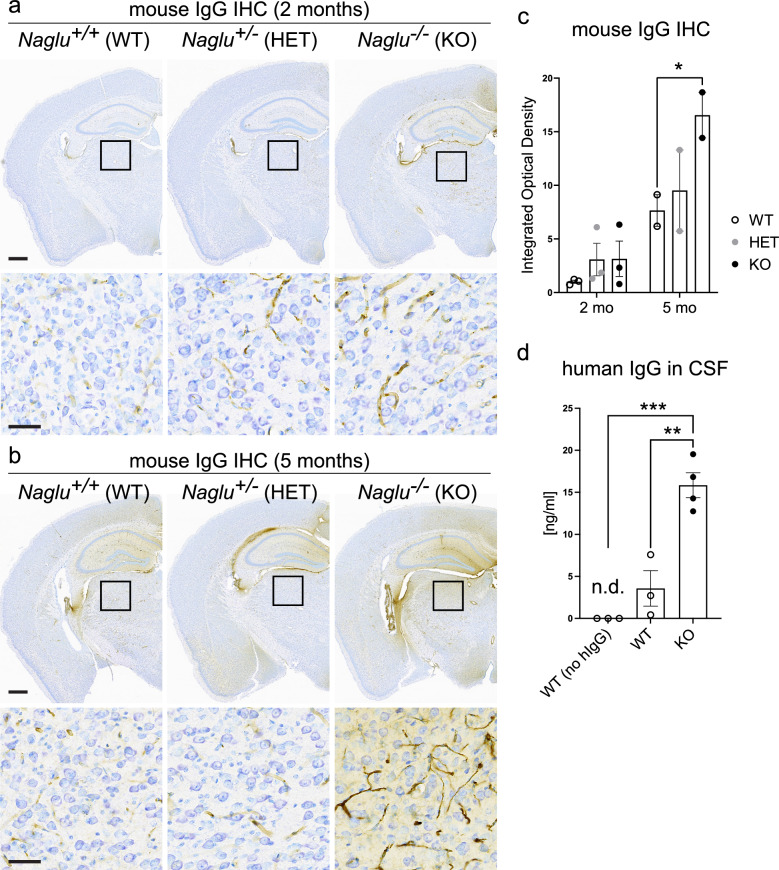
The blood–brain barrier deteriorates progressively in MPS IIIB mice. (**a**) *Top panel*: Images of brain coronal sections from 2-month-old *Naglu* KO and control animals stained for endogenous mouse IgG. Boxes indicate the areas depicted in the bottom panel images. Scale bar, 500 µm. *Bottom panel:* Close-ups from the images in the top panel magnifying areas in the thalamus demonstrating mouse IgG immunoreactivity within blood vessels. Scale bar, 50 µm. (**b**) *Top panel*: Images of brain coronal sections from 5-month-old *Naglu* KO and control animals stained for endogenous mouse IgG. Boxes indicate the areas depicted in the bottom panel images. Scale bar, 500 µm. *Bottom panel:* Close-ups from the images in the top panel magnifying areas in the thalamus demonstrating mouse IgG immunoreactivity within blood vessels and brain parenchyma in *Naglu* KO. Scale bar, 50 µm. (**c**) Quantification of mouse IgG IHC signal in coronal brain sections from 2- and 5-month-old *Naglu* KO and control animals (analyzed n = 3 sections/brain in 2–3 animals/group). Data shown as mean ± SEM; one-way ANOVA with Tukey’s post-hoc multiple comparisons test; *p < 0.05. (**d**) Human IgG ELISA showing amount of human isotype control antibody present in the CSF of 2-month-old *Naglu* KO and control animals at 48 h post-dosing, as well as in 2-month-old control WT animals that did not receive hIgG treatment. Data shown as mean ± SEM; one-way ANOVA with Tukey’s post-hoc multiple comparisons test; **p < 0.005; ***p < 0.0005; n.d. – not detected.

Previous studies have demonstrated significant structural and functional impairment of the blood–brain barrier (BBB) in *Naglu* KO mice at 3 months of age with the onset of early disease symptoms^33^. This is consistent with the vacuolations and storage material buildup in pericytes and perivascular spaces we and others observed in the brains of younger 1- to 2-month-old KO mice (Fig. 2b)^32^. In addition, as astrocytes are an important structural and functional component of the BBB^41,42^, the early onset of reactive astrogliosis in the KOs (Fig. 3c,d)^34,39^ is also likely to contribute to BBB leakiness. In order to assess the integrity of the BBB in younger *Naglu* KO brains, prior to the onset of symptomatic disease, we stained brain sections against endogenous mouse immunoglobulin G (msIgG) (Fig. 4a,b). It is expected that msIgG, along with other serum proteins, is more likely to pass into the brain parenchyma if the BBB has been compromised^43^. Indeed, we observed a notably higher mouse IgG IHC signal in *Naglu* KO brains at 2 months of age (Fig. 4a), which became significantly more enhanced by 4–5 months of age (Fig. 4a–c), indicating progressive deterioration in the BBB. The hippocampal and medial motor cortex area appeared to be most affected (Fig. 4a,b), consistent with previous reports^33^. Next, we tested the functional integrity of the BBB in 2-month-old *Naglu* KO mice. We dosed KO and littermate control animals with human IgG (hIgG) and 48 h post-dose analyzed the amount of human protein in the animals’ CSF. Interestingly, we observed a fourfold increase in hIgG in the CSF of KO mice (Fig. 4d). This suggests that deterioration of the BBB, or the blood-CSF barrier, resulting from loss of NAGLU function likely occurs well before the onset of symptomatic disease.

### Proteomic signatures of disease pathology in *Naglu* KO mice

**Figure 5:**
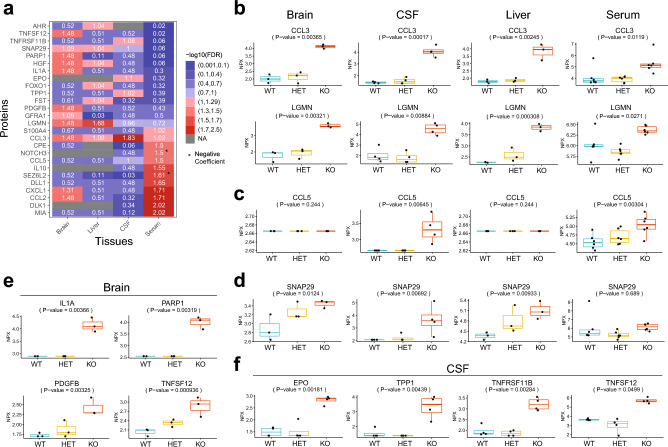
OLINK proteomic analysis of MPS IIIB mouse tissues. (**a**) Heat map of proteins with significant (adjusted FDR P < 0.1) changes in *Naglu* KO animal expression compared to controls in at least one of the matrices analyzed—serum, CSF, liver or brain tissue. (**b**) Box plots demonstrating significant upregulation in the expression of two proteins in KO animals across all tissues analyzed. (**c**) Box plots demonstrating differential upregulation in the expression of CCL5 in serum and CSF from KO animals. (**d**) Box plots demonstrating significant upregulation in the expression of SNAP29 in three tissue types in *Naglu* KO animals compared to controls. (**e**) Box plots showing significant changes in protein expression specifically in *Naglu* KO brain tissue. (**f**) Box plots showing significant changes in protein expression specifically in *Naglu* KO CSF.

Despite the monogenic nature of MPS IIIB, many of the complex signaling mechanisms that drive or are altered in the course of the disease remain unclear. To explore the landscape of proteomic changes occurring in *Naglu* KO mice we probed serum, cerebrospinal fluid (CSF), brain and liver tissues in mutant and control animals at 4–5 months of age. Using the Proximity Extension Assay-based Olink technology^44^ we analyzed the levels of a targeted subset of 92 proteins involved in key biological processes (Fig. 5 and Supplementary Table S1). We identified changes in the levels of 4 proteins that were significant specifically in *Naglu* KO brain tissue (GFRA1—GDNF family receptor alpha-1, IL1A—Interleukin-1 alpha, PARP1—Poly [ADP-ribose] polymerase 1, PDGFB—platelet derived growth factor subunit B) (Fig. 5a,e). Another 3 proteins were uniquely changed in the CSF of KO animals (EPO—Erythropoietin, TNFRSF11B—TNF receptor superfamily member 11b, TPP1—Tripeptidyl peptidase 1) (Fig. 5a,f). We also identified 3 proteins (AHR—Aryl hydrocarbon receptor, FST—Follistatin, FOXO1—Forkhead box protein O1) whose levels were significantly altered in *Naglu* KO liver tissue, whereas 7 proteins were differentially expressed in KO mouse serum only (CPE—Carboxypeptidase E, DLK1—Protein delta homolog 1, DLL1—Delta-like protein 1, IL10—Interleukin-10, MIA—Melanoma-derived growth regulatory protein, NOTCH3—Neurogenic locus notch homolog protein 3, SEZ6L2—Seizure 6-like protein 2) (Fig. 5a). SNAP29—Synaptosomal-associated protein 29, was the only protein significantly upregulated in three tissue types (Fig. 5a,d), whereas a number of others (CCL2—C–C motif chemokine 2, CCL5—C–C motif chemokine 5, CXCL1—Growth regulated alpha protein, HGF—Hepatocyte growth factor, Protein S100A4, TNFSF12/TWEAK—TNF ligand superfamily member 12) were significantly changed in at least two of the tissue types tested (Fig. 5a,c,e,f).

Most importantly, 2 proteins—CCL3 (C–C motif chemokine 3) and LGMN (Legumain) (Fig. 5a,b), were significantly upregulated across all matrices. CCL3/MIP1α is a cytokine produced primarily by monocytes and macrophages and is involved in microglial activation and the recruitment of peripheral inflammatory cells to the brain parenchyma. In addition to CCL3, another chemokine—CCL5, which could be a potential biomarker in human MPS IIIB plasma^45^, was also significantly upregulated in both *Naglu* KO mouse CSF and serum (Fig. 5a,c). Consistent with this, the DAVID (GO BP and KEGG) pathway analysis we performed further pointed to immune response and cytokine receptor activation signaling as most enriched in *Naglu* KO CNS (Supplementary Fig. S1 and Supplementary Tables S2 and S3).

The cysteine protease LGMN, which was also upregulated in all analyzed KO tissue types (Fig. 5a,b), is thought to be involved in the processing of pathogen-derived and endogenous proteins for MHC class II presentation and is required for normal lysosomal protein degradation^46,47^. Additionally, the significant changes we observed in the levels of SNAP29 (Fig. 5a,d)—a soluble N-ethylmaleimide-sensitive factor-attachment protein receptor (SNARE), which plays a role in the membrane fusion of autophagosomes with the lysosome^48^, may further point to an uptick in autophagy in *Naglu* KO mouse tissues.

Among the CNS-specific proteins upregulated in *Naglu* KO brains was the GDNF receptor GFRA1 (Fig. 5a), which suggests an upregulation in the neuroprotective GDNF signaling pathway^49^. We also noted significant increase in interleukin IL1A, as in previous studies^50^, and PARP1 levels (Fig. 5e), consistent with chronic brain inflammation in MPS IIIB mice (Figs. 3c–g, 5b,c).

PDGFB, also significantly upregulated in *Naglu* KO brains (Fig. 5e), is essential for maintaining BBB integrity both during development and in adulthood^51–53^, and upregulation in PDGFB signaling has previously been reported following cerebral ischemia^54^. Additionally, the cytokine TNFSF12/TWEAK, which was increased both in *Naglu* KO brain tissue and CSF (Fig. 5e,f), is known to actively promote BBB disruption and neural cell death^55^, and could be a likely contributor to the progressive BBB degradation in *Naglu* KO brains. Interestingly, in addition to altered cytokine signaling, our DAVID (KEGG) pathway analysis based on significant proteomic changes we observed in *Naglu* KO brains also implicated the mitogen-activated protein kinase (MAPK) signaling pathway (*p* = 0.0004) (Supplementary Fig. S1 and Supplementary Table S2).

The three proteins we found uniquely upregulated in *Naglu* KO mouse CSF (Fig. 5f) span a wide range of functions and signaling processes. TNFRSF11B, normally involved in bone homeostasis, can be expressed in brain glial and neuronal cells in certain ischemic stress conditions, indicating a role in brain injury following inflammation^56,57^. It remains unclear, however, whether its role in ischaemia and MPS IIIB is a neurotoxic or a protective one. EPO, another protein that we found significantly upregulated in *Naglu* KO CSF, also plays a positive role in brain development and neuroprotection^58^. Additionally, we detected a significant increase in TPP1—a lysosomal enzyme, which is responsible for the cleavage of N-terminal tripeptides from a wide range of substrates and is absent in a subtype of late-infantile neuronal ceroid lipofuscinoses^1,2^.

### Minor behavioral deficits observed in *Naglu* KO mice

**Figure 6:**
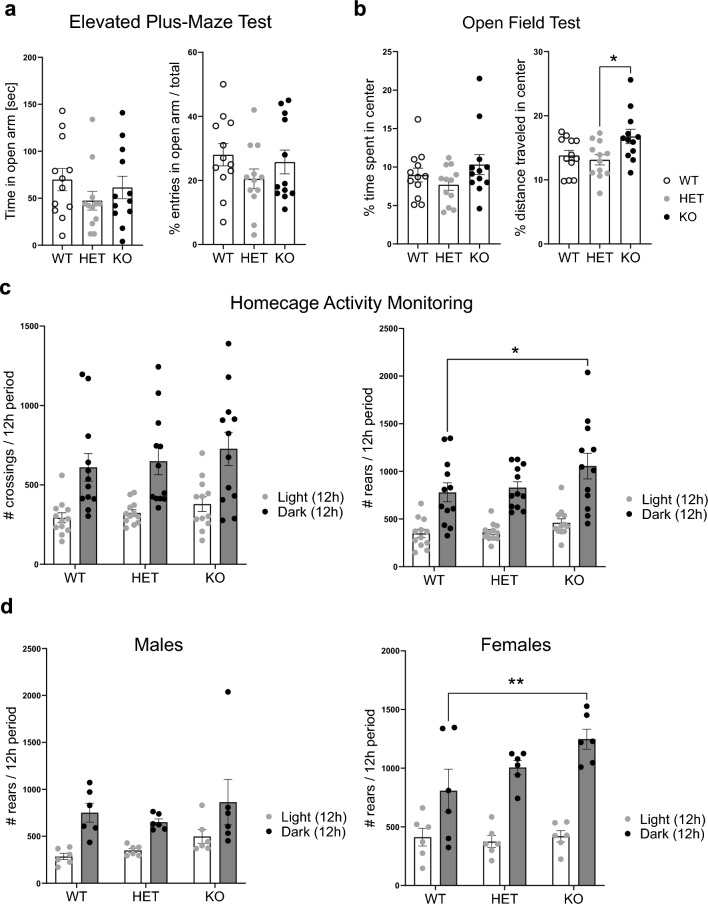
*Naglu* KO mice exhibit hyperactive behavior. (**a**) Results from elevated plus-maze behavioral tests performed on 4- to 5-month-old *Naglu* KO and control animals (n = 12 animals/ group, mixed-sex). Plots show the amount of time each group spent in the open arm of the maze (*left*), and the fraction of entries into the open arm (*right*), during the 10-min course of the test. Data shown as mean ± SEM; no significant differences were detected by one-way ANOVA with Tukey’s post-hoc multiple comparisons test. (**b**) Open field test in the same group of animals from **a**) (4- to 5-month-old *Naglu* KO and control animals; n = 12/group, mixed-sex). Plots show the fraction of time each group spent in the 20 cm x 20 cm center zone of the field as opposed to in the periphery (*left*), and the fraction of total distance covered while in the center zone (*right*), during the 5-min course of the test. Data shown as mean ± SEM; ordinary one-way ANOVA with Tukey’s post-hoc multiple comparisons test; *p < 0.05. (**c**) Homecage activity monitoring was performed on 4 to 5-month-old *Naglu* KO and control animals (n = 12/group, mixed-sex) over the course of 24 h. The number of mark-crossings (*left*) and number of rears (*right*) for each group were plotted during the 12 h of light and 12 h of active dark period, respectively. Data shown as mean ± SEM; ordinary one-way ANOVA with Tukey’s post-hoc multiple comparisons test; *p < 0.05. (**d**) Number of rears performed by the male (n = 6/group, *left*) and female-only (n = 6/group, *right*) mice in the cohort during the homecage activity monitoring test in (**c**). Data shown as mean ± SEM; ordinary one-way ANOVA with Tukey’s post-hoc multiple comparisons test; **p < 0.001.

Earlier studies note the first signs of significant behavioral defects in *Naglu* KO mice after 4 months of age, which marks the onset of the symptomatic stage of the disease in mice^30,31^. Behavioral abnormalities continue to exacerbate with age in this genetic model of MPS IIIB and ultimately give way to general locomotor defects^31,32,34^. To validate further the pathological deficits of the *Naglu* KO mouse model, we exposed a mixed-sex cohort of 4- to 5-month-old *Naglu* KO and littermate control animals to a series of anxiety-measuring tests. When analyzing the time naïve animals spent in a more exposed, anxiogenic, area of the test field during Elevated-Plus Maze and Open Field tests (Fig. 6a,b), we observed no significant difference in each group’s pattern of behavior. Both control and *Naglu* mutant animals showed a strong preference towards avoiding the exposed areas of the test field. However, there was a notable increase in the distance covered by KO mice when in the center of the Open Field (Fig. 6b), suggesting an elevated state of activity consistent with previous reports^32^.

We next analyzed the overall activity levels of individually housed *Naglu* KO and littermate control mice over a 24-h period via the Homecage Activity Monitoring Test (Fig. 6c,d). As nocturnally active animals, both control and KO mice were substantially more active during the 12 h dark cycle of the test. Notably, *Naglu* KO mice demonstrated a significantly greater increase in nocturnal activity compared to control groups (Fig. 6c), as measured by the number of rears recorder in KOs. These results, along with previous reports^32^, indicate hyperactive behavior in NAGLU-deficient mice, which is consistent with some of the clinical symptoms observed in MPS IIIB pediatric patients. Interestingly, this behavior was more pronounced in female animals (Fig. 6d), implying the presence of sexually dimorphic behavioral features in this mouse model. Overall, based on the applied tests, *Naglu* KO animals appear to exhibit only relatively mild deficits in behavior several months after the onset of significant histopathological signs of neurodegeneration and neuroinflammation (Fig. 3).

## Discussion

In this study, we attempted to provide a more comprehensive overview of the *Naglu* KO mouse model of MPS IIIB in order to identify key signature pathological processes that could be applicable to the human condition and to future therapeutics development. In addition to uncovering evidence for the presence of hepatomegaly in *Naglu* KO mice, which is reminiscent of a key symptom in patients^25^, we were also able to confirm earlier findings demonstrating the presence of widespread chronic neuroinflammation and BBB disruption in the CNS of this animal model. The BBB plays an essential role in maintaining brain homeostasis by regulating the exchange of molecules between the body’s systemic compartment and the brain parenchyma^41,42^. The structural and functional integrity of the BBB protect the brain from pathogens and other harmful molecules, hence BBB leakiness could be a key driver of the neuroinflammatory and other pathological processes in MPS IIIB. The extent of BBB impairment in MPS IIIB patients remains unclear. However, if the degree of BBB damage in Sanfilippo IIIB patients is similar to that observed in other MPS III subtypes^59^ or if it bears any resemblance to the *Naglu* KO mouse model, it would be important to understand the clinical aspects of whether and how BBB compromise in MPS IIIB affects disease pathogenesis and progression. This should also come into consideration in the context of developing enzyme replacement therapies (ERT), particularly when testing in the *Naglu* KO MPS IIIB mouse model, as BBB leakiness might affect the biodistribution and/or efficacy of novel therapeutics.

Innate immune cells, such as microglia and macrophages, have been shown to actively contribute to LSD progression by releasing cytokines and chemokines, thus further amplifying the initial inflammatory response triggered by dying and/or damaged neurons^60^. Our histological (Fig. 3) and proteomic data (Fig. 5a–c), in addition to previous findings in mice^34–37^ and humans^45^, provides strong evidence for the damaging neuroinflammatory effects of NAGLU loss-of-function in this MPS IIIB mouse model. Albeit limited in its capacity, our proteomic analysis of *Naglu* KO mouse tissues (Fig. 5) helps to paint a complex picture of disease pathology, which appears to emerge at the convergence of many factors. The inactivation of NAGLU—a single lysosomal enzyme, triggers the progressive accumulation of its primary GAG substrate—HS, which then appears to exert a direct effect on the levels of inflammatory factors (CCL3, Fig. 5b) and enzymes (LGMN and TPP1, Fig. 5b and f, respectively). Previous studies have shown elevated CCL3 in the serum^61^ and brains^50^ of MPS IIIB mice, as well as significant increase in *Ccl3* transcription in 10-day-old MPS IIIB mouse cortices and in MPS IIIB microglial cultures following in vitro stimulation with HS^37^. At earlier stages of MPS IIIB in mice, this appears to be a direct result of microglial priming by HS oligosaccharides binding to Toll-like receptor 4 (TLR4)^37^, which operates upstream of CCL3 and is a known receptor for HS in immune cells^62–64^. In addition, a number of GAGs, including HS, can stimulate the catalytic autoactivation of LGMN^65^, which in turn can induce changes in interleukin levels^66^. Consistent with its known functions and given the increase in lysosomal HS accumulation in MPS IIIB, the observed increase in LGMN levels in *Naglu* KO mouse tissues is also likely a direct result of GAG buildup and reflects a general disruption in normal lysosomal function in the absence of NAGLU. The resulting dysregulation of lysosomal function, particularly in microglia and macrophages, is apparent from both the increased expression of inflammatory factors (Fig. 5a–c,e) and the enlarged lysosomal compartments (Fig. 2b) and morphological changes in *Naglu* KO microglial cells (Fig. 3e). Similar to other lysosomal enzymes, TPP1 is first synthesized as a catalytically-inactive pro-enzyme and is activated later upon acidification. Both dermatan sulfate and HS can promote TPP1 pro-enzyme activation but inhibit the activity of the mature enzyme in a dose-dependent fashion^67^. This upregulation of TPP1 in *Naglu* KO CSF is likely a direct result of HS and/or other secondary ganglioside accumulation in MPS IIIB^68^, and is a further indication of a disrupted autophagosome-lysosomal function in this animal model.

Although it has been suggested that the widespread neuronal degeneration in MPS IIIB (Fig. 3a,b) occurs independently from the HS-driven priming and activation of microglia^37^, the exact mechanisms of initiation of neuronal demise remain unclear, particularly at early stages of the disease. The upregulation of both neuroprotective (GFRA1, PDGFB, EPO—Fig. 5a,e,f) and neuro-disruptive factors (IL1A, PARP1, TNFSF12, TNFRSF11B—Fig. 5e,f) we observed in the CNS of *Naglu* KO mice by 5 months of age, could be either a trigger and/or outcome of neural cell death. The changes in PDGFB levels we detected in *Naglu* KO mouse brains are consistent with and likely triggered by the degradation in BBB integrity we observed in young KO mouse brains (Fig. 4). Increase in PARP1, which recognizes DNA breaks and actively recruits repair factors^69^, could potentially be a result of the neuronal damage and cell death present at early stages in the brains of *Naglu* KO mice (Fig. 3a,b). On the other hand, as PARP1 plays a pleiotropic role in a range of cellular processes, including the induction of an inflammatory response^69^, increase in the levels of this protein might itself constitute one of the triggers for the widespread brain inflammation in *Naglu* KO mouse brains.

The upregulation of pro-inflammatory factors we and others^50,61^ have detected both in the brain and peripheral tissues of *Naglu* KO mice provides strong evidence for inflammation being a major driver of the pathological process in the early stages of MPS IIIB. This notion is also supported by a report demonstrating that prednisolone—an immunosuppressant and anti-inflammatory, can improve *Naglu* KO mouse behavior, presumably by reducing peripheral cytokine levels but without having an effect on brain pathology^50^. With this in mind, anti-inflammatory therapies could prove beneficial in alleviating some of the symptoms or staving off disease progression in pediatric MPS IIIB patients, as has been demonstrated in a MPS IIIB mouse model^70^ and in other LSDs^60,71^. Proteomic analysis of 8-month-old *Naglu* KO mouse brains using tandem mass spectrometry coupled with liquid chromatography also uncovered significant changes in cytoskeletal, metabolic and synaptic vesicle trafficking processes in KOs compared to healthy controls^72^. Altogether, these findings suggest that while immune and inflammation dysregulation is a prominent hallmark early in the disease process, the MPS IIIB pathological landscape changes over time. A broader and more systematic approach using diverse proteomic analysis methods to assay a wider variety of proteins in different tissue types from *Naglu* KO mice would be beneficial in identifying novel tissue- and age-specific biomarkers of MPS IIIB. In addition, the secondary accumulation of gangliosides such as GM2 and GM3 in MPS IIIB and other LSDs, which has been extensively discussed elsewhere^68^, is another contributing factor to MPS IIIB pathology whose mechanism needs further exploration.

Some of the early signs associated with MPS III include behavioral deterioration, hyperactivity and autism spectrum disorder (ASD)-like symptoms in the first years of life^26^. Despite the severe biochemical and early histopathological deficits we observed in 2-month-old *Naglu* KO mouse brains, our anxiolytic behavior testing in 4–5-month-old mutant and control mice revealed no major deficits with the exception of increased hyperactivity predominantly in female *Naglu* KO animals (Fig. 6c,d). Further deterioration in behavior and motor function has been reported in aged (> 6-month-old) *Naglu* KO animals^31,32,34^, although to what extent these changes match the severity of mental decline and dementia of MPS IIIB patients is unclear. Our CNS proteomics-based pathway analysis pointed to dysregulation in MAPK signaling (Supplementary Fig. S1). Precise regulation of this pathway is essential for normal neural development and disruption in MAPK signaling is known to result in neurodevelopmental disorders such as ASD^73^. Although ASD-like behaviors have been reported in other MPS mouse models, such as MPS II and IIIA^74,75^, whether such cognitive changes are present in *Naglu* KO mice remains largely unexplored. Altered dopaminergic signaling driven by the HS dysfunction typical for MPS has been identified as a main cause of ASD in MPS IIIA mice^75^. However, it remains to be determined whether that is the case in *Naglu* KO mice as well. Further analysis testing the social, communicative and perseverative behaviors of MPS IIIB mice^76^ will be necessary to fully characterize the cognitive state of this mouse model.

Our study builds on earlier reports and further illustrates the presence of substantial and likely irreversible neuropathology in young asymptomatic *Naglu* KO mice. Due to the vulnerability of the MPS IIIB pediatric patient population and the developmental etiology of the disease, it is increasingly important to utilize the *Naglu* KO model to gain a better insight into the embryonic and early postnatal stages of disease onset and progression. Despite some success in preclinical models^24,29^, there are still no therapies for MPS IIIB that successfully resolve neurological symptoms in patients, prompting the need for initiating treatment early in development (prenatally). To this end, the very recent success of the first reported in utero ERT treatment for infantile Pompe disease^77^, offers an encouraging new route for treatment of these debilitating neurodevelopmental LSDs. In utero ERT, in combination with improved carrier screening or prenatal diagnostics, could provide the much-needed breakthrough in curing LSDs and circumvent the need for developing therapeutics able to cross the BBB—a common challenge with all current ERTs for LSDs affecting the CNS.

## Materials and methods

### Animals

All methods used in this study were performed in accordance with the national and international recommendations for the care and ethical use of laboratory animals. *B6.129S6-Naglutm1Efn/J* (*Naglu*^*-*^, Stock #003827)^30^ mouse strain was obtained from The Jackson Laboratory (JAX), where the line was cryorecovered by using sperm from Stock #003827 male mice and oocytes from C57BL/6J (Stock #000664) female mice. The resulting heterozygous mice were intercrossed to establish a colony for dedicated supply. Animals were bred and genotyped at JAX using established protocols (JAX Protocol 28548). Procedures for this study were carried out in accordance with the Guide for the Care and Use of Laboratory Animals (2011) and under approval of the Institutional Animal Care and Use Committees (IACUC) at JAX. All animal studies and methods used that we report here comply with ARRIVE guidelines and with the IACUC-approved animal-use protocols at the contract research organizations where testing took place (JAX, Charles River Laboratories and Porsolt, respectively—all AAALAC accredited sites). Animals were assigned to study groups based on genotype: wild-type (WT; *Naglu*^+*/*+^), heterozygotes (HET; *Naglu*^+*/−*^), and homozygous/knockout animals (KO; *Naglu*^*−/−*^), with equal representation of both sexes in each group and blinded experimental setup enforced whenever reasonably possible.

### Pathological studies

Tissue processing for biochemical and histological analysis was performed at Charles River Laboratories, Worcester, MA. All experiments were carried out at a facility approved and accredited by the Association for Assessment and Accreditation of Laboratory Animal Care (AAALAC) International, and within the approved global protocols set forth and governed by the Charles River Laboratories IACUC Committee and in accordance with all USDA, FDA, AAALAC, and *The Guide* regulations governing any and all industry standards and regulations for the execution of In Vivo studies. Upon arrival, the animals were acclimated to the Test Facility for at least 2 days prior to study start. Both male and female *B6.129S6‐Naglutm1Efn/J* mice were used in this study. Body weights were recorded upon arrival and once weekly throughout the study. All animals were monitored for the duration of the study and all abnormalities were recorded—moribund animals were terminated following a terminal blood collection with veterinarian approval.

Serial blood samples were collected via tail vein snip or facial venipuncture in accordance with the test facility’s standard operating procedures. Terminal blood samples were collected at the designated timepoint via cardiac venipuncture following inhalation anesthesia in accordance with test facility standard operating procedures. Blood samples were collected into tubes with appropriate additive and the tubes were stored at ambient temperature for at least 30 min to allow for clotting until processed to serum by centrifugation (3500 rpm at 5 °C for 10 min) within 1 h of collection then stored at − 80 °C. Immediately following blood sample collection, CSF samples were collected into appropriately sized pre-weighed tubes. Individual CSF weights were calculated and CSF samples were stored at − 80 °C until transferred for analysis. For cryopreserved tissue, following blood and CSF collection, the animals were perfused intracardially with 50 mL of normal saline at a rate of 10 mL/min. Immediately following saline perfusion, brain and liver samples were collected and placed into individual uniquely labeled pre-weighed 20 mL disposable vials—organ weights were calculated and recorded for each sample. The vials were then flash frozen on liquid nitrogen and stored at − 80 °C until analysis. For histological preparations, following blood, CSF collection and saline perfusion, animals were perfused again with 50 mL of 4% paraformaldehyde (PFA) solution at a rate of 10 mL/min. After the perfusion, the brain and liver were carefully removed and post-fixed in a 4% PFA solution for 24 h at 2–8 °C. The tissue was then transferred into new 20 mL vials containing PBS and stored at 2–8 °C until further analysis.

### NAGLU fluorometric endpoint enzyme activity assay

Saline-perfused and flash-frozen brain tissue from 2-month-old *Naglu* KO and wild-type littermate control animals (n = 2/genotype) was collected as described above and lysed in N-PER™ Neuronal Protein Extraction Reagent (Thermo Scientific, Cat. #87792) without protease inhibitors. Lysates were further freeze-thawed several times and briefly centrifuged to remove large debris. Protein sample concentrations was measured and normalized with N-PER buffer. For the enzymatic reaction, 10 µL of 2 mg/mL or 18 mg/mL brain sample lysate were combined with 5 µL of 20 mM 4-methylumbelliferyl N-acetyl-α-d-glucosaminide substrate (MilliporeSigma, Cat. # 474500) in 35 µL of 100 mM sodium acetate buffer pH 4.6. The reaction was assembled in a 96-well plate and incubated at 37 °C for 3 h. Fluorescence standard curves were produced using 4-methylumbelliferone (Thermo Fisher Scientific, Cat. #AAA103370B) stocks in methanol, diluted with sodium acetate buffer pH 4.6 on the same reaction plate. Enzymatic reactions were stopped by adding 100 µL of glycine buffer pH 10.3 to each sample and standard well. Fluorescence signal was immediately measured on FlexStation3 microplate reader (Molecular Devices LLC, San Jose, CA, U.S.A) using the following wavelengths: λ_ex_ 365 nm; λ_em_ 460 nm.

### Human IgG ELISA

*Naglu* KO and wild-type littermate control animals (n = 3–4/group) were dosed at 30 mg/kg i.v. at ~ 2 months old with control human IgG1 3D5 antibody^78^ produced in-house. Dosed animals were sacrificed 48 h post-treatment and terminal CSF was collected as described. The concentrations of human IgG in murine CSF were measured with a human IgG ELISA kit (Abcam, Cat. #ab212169). An 8-points standard was prepared by diluting the kit-provided Human IgG protein to 15 ng/mL followed by a 1:2 serial dilution. Standards and test samples were diluted so that the antibody concentration was below 15 ng/mL and loaded at 50 µL/well onto anti-tag immobilization coated 96-well plates, and topped with 50 µL of antibody cocktail containing a mixture of capture and detector antibody Plates were washed and TMB was added as a chromogen at 100 µL/well. Sealed plates were incubated shaking at room temperature for 5 min to develop the color reaction. The intensity of the color reaction was assessed by absorbance at a wavelength of 450 nm. Sample protein concentrations were determined by interpolating the blank control absorbance values subtracted against the respective antibody standard curve with a second order polynomial model.

### Electron microscopy

For ultrastructural analysis of brain and liver tissue, 2- and 4-month-old *Naglu* KO, heterozygous and wild-type littermate control female mice (1 animal/group) were processed at Charles River Laboratories. The animals were perfused intracardially with 10 mL of room-temperature saline using a 21G needle and then with 10 mL of Modified Karnovsky's fixative (Electron Microscopy Sciences, Cat. # 15720, Mixture 2). Upon perfusion, the brain and liver were removed and carefully trimmed (motor cortex area was used for brain analysis) into cube(s) measuring 1 mm × 1 mm × 1 mm, which were placed into 3–4 mL vials with Modified Karnovsky’s fixative and refrigerated overnight. The following day samples were processed through to four resin blocks per tissue per animal. All blocks were thick-sectioned at approximately 0.5–1 µm thickness and stained with toluidine blue. Slides were scanned to determine the best area per sample for thin sectioning. Thin sections were then prepared from the most optimal tissue block and examined on a JEOL JEM 1400+ Transmission Electron Microscope. All digital images were captured using the AMT XR16MP digital camera system and made available to a board certified CRL veterinary pathologist for ultrastructural microscopic evaluation. Approximately ten digital electron micrographs of each brain and liver sample were evaluated at direct magnifications ranging from 400× to 6000× (printed images viewed at 2100× to 31600×).

### Immunohistochemistry

Neurohistology was performed at NeuroScience Associates, Inc. The brains were examined and then cryopreserved overnight with 20% glycerol and 2% dimethyl sulfoxide in PBS. The specimens were then embedded, with up to 25 mouse brains per block, arranged for coronal sectioning in a gelatin matrix using MultiBrain^®^ Technology (NeuroScience Associates, Knoxville, TN). After curing with a formaldehyde solution, the blocks were rapidly frozen by immersion in 2-methylbutane chilled with crushed dry ice and mounted on a freezing stage of an AO 860 microtome. The MultiBrain^®^ blocks were sectioned coronally at 35 µm. Sections were collected sequentially into a series of 24 cups and stored in Antigen Preserve solution (50 parts PBS pH7.0, 50 parts ethylene glycol, 1 part polyvinyl pyrrolidone) until analyzed. Free-floating IHC was performed on every twelfth section (at an interval of 420 µm). All incubation solutions from the primary antibody onward as well as all rinses used Tris buffered saline (TBS) with Triton X100 (Promega, Cat. #H5141). Following hydrogen peroxide treatment and rinses, sections were immunostained overnight at room temperature with the following primary antibodies: rabbit anti-GFAP (Dako, Cat. # Z0334); rabbit anti-IBA1 (Abcam, Cat. # ab178846); rabbit anti-NAGLU (Atlas Antibodies, Cat. #HPA038815). Following rinses, a biotinylated secondary antibody (goat anti-rabbit, Vector Labs, Cat. # BA-1000, Burlingame, CA) was applied. After further rinses VECTASTAIN^®^ Elite ABC solution (Vector Labs, Cat. # PK-6100) was applied at a dilution of 1:222. The sections were again rinsed, then treated with a chromagen: diaminobenzidine tetrahydrochloride (DAB) and hydrogen peroxide to create a visible reaction product. The chromagen for the IBA1 and NAGLU IHC included nickel (II) sulfate. In order to detect endogenous mouse IgGs and test for BBB permeability, brain sections were immunostained directly with biotinylated horse anti-mouse IgG secondary antibody (Vector, Cat. # BA-2001), and then stained with a Light Thionine Nissl counter stain. Stained sections were mounted on gelatin-coated glass slides, air dried, then dehydrated in alcohols, cleared in xylene and coverslipped with Permount (Fisher Scientific, Pittsburgh PA).

Amino Cupric Silver Stain was performed on free-floating sections using the de Olmos amino cupric silver (AmCuAg) method^38^, where silver ions form complexes with the exposed amino acid chains from the denatured proteins in degenerating neurons, which then appear as black-stained structures. Briefly, the method includes the following major steps: Incubation in Pre-Impregnation solution (cupric nitrate, silver nitrate, cadmium nitrate, lanthanum nitrate, neutral red, alpha-amino butyric acid, alanine, pyridine, triethanolamine, isopropanol and dH2O) in a ~ 47 °C water bath; Impregnation (100% ethanol, formaldehyde, citric acid and dH2O) at room temperature; Reduction (100% ethanol, formaldehyde, citric acid and dH2O) in a 32–35 °C water bath; Bleaching in potassium ferricyanide in potassium chlorate with lactic acid, and potassium permanganate with weak sulfuric acid; and finally Fixing in sodium thiosulfate. During the reduction stage, the cupric ions function as an external reducer for the silver ions against the natural properties of the tissue thus creating greater contrast between normal tissue and damaged neuronal structures. The stained sections were then cleared in Kodak Rapid Fixer Solution, mounted, air dried and counterstained with Neutral Red. All stained slides with ~ 25 mouse brain sections on each were then imaged at 20× on a Huron Digital Pathology Whole Slide Imaging system.

### Image analysis

High-resolution TIFF images of stained 25-brain-sections slides were analyzed on HALO™ (v3.2, Indica Labs, Albuquerque, NM, USA). For the image analysis, a minimum of 3 rostral to caudal coronal brain sections were selected from each animal, which were also matched based on gross anatomical landmarks. Images of sections from 2–3 animals were analyzed for each condition. IHC analysis was performed on the entire coronal brain section in order to include multiple brain regions. The HALO tissue classifier module was applied to the images, to separate the tissue in the image from non-tissue areas (i.e. glass) based on an artificial intelligence random forest algorithm^79^. The Area Quantification module of HALO was used to measure the intensity of the IHC staining within the tissue portion of the selected brain section images. Following HALO analysis completion, the ‘Integrated Optical Density (OD)’ for each imaged section was calculated as the product of the average IHC intensity across all pixels in the section (optical density) multiplied by the percentage of the chromogen-stained area. The average value for the ‘Integrated OD’ of all section images for each analyzed brain was used to compare IHC staining between groups of animals. To quantify the number of IBA1-expressing cells, the HALO Object Colocalization module was applied instead following Tissue-Glass classification, allowing the identification of stained cells and ‘counting’ them as objects based on size, morphology, IHC staining intensity, etc. excluding diffused non-cellular staining in the process. The average number of IBA1+ objects (cells) identified per mm^2^ section area was then plotted for each analyzed brain. Quantified IHC data was graphed and analyzed in Prism 9 software (GraphPad Software, Inc., La Jolla, CA, U.S.A).

### Proteomic analysis

#### Olink assay

Serum (n = 6/genotype) and CSF (n = 4/genotype) samples collected from *Naglu* KO, heterozygotes and wild-type littermate controls at ~ 4–5 months of age as described above, were analyzed using the Olink® Target 96 Mouse Exploratory Assay (Product number: 95380). In addition, 1 mg/mL protein lysates from flash-frozen brain and liver tissue samples (n = 3/genotype for each matrix) collected from 4–5-month-old mice were prepared in T-PER™ Tissue Protein Extraction Reagent (Thermo Scientific, Cat. #78510) with added protease inhibitors (1 mM PMSF, 10.4 mM AEBSF, 8 μM Aprotinin, 0.2 mM Leupeptin, 0.4 mM Bestatin, 0.15 mM Pepstatin, 0.14 mM E-64) according to manufacturer’s guidelines for alternative matrices. All samples were then shipped to Olink Proteomics Inc (Waltham, MA) for assay execution and data collection. Results from the analyzed protein biomarkers was then provided in Normalized Protein eXpression (NPX) units.

#### Statistical analysis

Statistical analysis of Olink NPX data were performed in R (Statistical computing programming language^80^). We used the R package OlinkAnalyze^81^ for loading the NPX data. The data manipulations were performed using dplyr^82^, tidyr^83^, reshape2^84^, and stringr^85^ packages. Lastly, the boxplots and heatmaps were generated using ggplot2^86^, ggpubr^87^, and gridExtra^88^ packages. The Olink data consisted of the normalized protein expression value (NPX), the limit of detection (LOD), and the missing data frequency for each protein represented by UNIPROT ID. We mapped the UNIPROT IDs to gene symbols using the ID mapping tool (https://www.uniprot.org/id-mapping) for downstream pathway analysis. Each sample was labelled as Wildtype (WT), Heterozygote (Het), or Knockout (KO). There were nine samples in Brain and Liver tissues, with three samples each in WT, Het, and KO groups. In CSF tissue, there were twelve samples, with four present in WT, Het, and KO groups. Finally, in Serum tissue, there were eighteen samples, of which each group had 6. There were 92 target mouse proteins included in each tissue panel: https://olink.com/content/uploads/2021/09/1082-v1.0-mouse-exploratory-panel-content-final.pdf. Missing data imputation was performed by assigning the missing value with the corresponding protein-specific LOD value.

For each tissue, we converted the NPX values to log10 (NPX) and then applied the linear regression method by coding the three genotype groups as WT = 0, Het = 1, and KO = 2 to obtain P-values for each protein. We created boxplots showing the average NPX expression and the P-values for each protein across different groups. Multiple testing adjustment was performed within each tissue using False Discovery Rate (FDR). Proteins with FDR < 0.1 in at least one of the tissues were included in the final heatmap. Analysis details are available in Supplementary Table S1.

#### Pathway analysis

We performed the pathway enrichment analysis using the database for annotation, visualization, and integrated discovery (DAVID, https://david.ncifcrf.gov/home.jsp, version 6.8, accessed on 01 December 2022) which is a publicly available bioinformatics database^89^ and Pathway studio which is commercial software from Elsevier^90^.

The DAVID database uses various functional annotation tools to identify the biological pathways of significantly expressed proteins. We retrieved the Gene ontology (GO, http://geneontology.org/, accessed on 01 December 2022) and Kyoto Encyclopedia of Genes and Genomes (KEGG, https://www.genome.jp/kegg/, accessed on 01 December 2022) results. The GO classifies the description of the gene into three main categories: biological process (BP), cellular component (CC), and molecular function (MF). The KEGG database provides an understanding of biological signaling pathways. We used the significant proteins (P-value < 0.05) obtained from each tissue (Brain, Liver, Serum, and CSF) along with the *NAGLU* KO gene as input. In each tissue comparison, the GO terms and KEGG pathways with P-value < 0.05 obtained from DAVID were considered to be statistically significant. DAVID pathway analysis details for Brain and CSF are available in Supplementary Table S2 and Supplementary Table S3, respectively.

Pathway analysis was conducted in Pathway Studio (PS) by connecting proteins with significant differential expression between genotypes in each tissue and known Mucopolysaccharidosis type IIIB –relevant pathways and concepts available in Elsevier Biology knowledge graph available from PS. The number of literature references along with the exact sentences curated by PS were reviewed for each connection. Additionally, novel pathways were built by manually assessing various relationships between OLINK proteins and Mucopolysaccharidosis type IIIB disease-relevant cellular processes and signaling transduction pathways. These analyses identified TLR4/CCL3, heparan sulfate (HS) and LGMN, TPP1 as most promising differentially expressed disease-related proteins in the associated pathways.

### Behavioral analysis

Behavioral analyses were performed at Porsolt Research Laboratories, France (AAALAC accreditation 2021). All animal studies were approved by the Porsolt Comité d'éthique n°60 and carried out in accordance with the Council Directive No. 2010/63/UE of September 22, 2010 on the protection of animals used for scientific purposes and French decree No. 2013-118 of February 1, 2013 on the protection of animals. 6 male and 6 female mice at 4–5 months old were included in each study group of *Naglu* KO, heterozygotes and wild-type littermate controls. Results were plotted and analyzed in Prism 9 software (GraphPad Software, Inc., La Jolla, CA, U.S.A).

#### Elevated plus-maze test

This method for detecting anxiolytic activity was originally described by Handley and Mithani^91^. The maze consisted of 4 arms of equal length and width (14 × 5 cm) arranged in the form of a plus sign (+). Two opposite arms were enclosed by 12 cm high walls (closed arms). The 2 other arms had no walls (open arms). The maze was raised approximately 60 cm above the floor. Each mouse was placed in the center of the plus-maze and left to explore for 10 min. The number of entries into the open and closed arms and the time spent on the open arms were recorded for a 10-min period. The percentage of open arm entries (open arm entries/total arm entries × 100) was calculated.

#### Open field test

Animals were placed in the center of a rectangular open field arena made of grey opaque Plexiglas (40 × 40 × 40 cm). The time spent and the distance moved in the periphery and the central zone (20 × 20 cm) and number of transitions between the two zones during a 5-min test were measured automatically using a video tracking system (EthoVision, Noldus). The percentage of time spent and distance moved in the central zone was also calculated. The test was performed in attenuated lighting conditions (30 lx at the center of the open-field).

#### Activity meter test

This method detects stimulant or sedative activity as originally described^92^. The activity meter consisted of 24 covered Plexiglas cages (20.5 × 10.5 × 18 cm) contained within a darkened cabinet and connected to silent electronic counters. Each cage was equipped with four photocell assemblies (two at each end of the cage) 2.5 cm above the floor, in order to measure the number of movements by each animal (one per cage) in the horizontal plane. Seven additional photocell assemblies were placed at even intervals 9.5 cm above the floor along the long wall to record rearing. The number of (horizontal) crossings by each animal (one per cage) from one pair of photocells to the other was recorded by computer in 60-min intervals for 24 h (12 h:12 h light/dark). A similar procedure was utilized for recording of rearing, except that individual photobeam breaks were recorded.

### Supplementary Information

Supplementary Table S1.

Supplementary Figure S1.

Supplementary Table S2.

Supplementary Table S3.

Supplementary Legends.

## Data Availability

Proteomic data generated and analyzed during this study are included as supplementary information files (Additional files 1, 2, 3, 4). Other original data files used in the study are available from the corresponding authors upon reasonable request.
