# Supplementary material for: Disease pathology signatures in a mouse model of Mucopolysaccharidosis type IIIB
Source: Sci Rep. 2023 Oct 4;13:16699. doi: 10.1038/s41598-023-42431-4 (PMC10550979; doi:10.1038/s41598-023-42431-4)

# DAVID Pathway Analysis - BRAIN

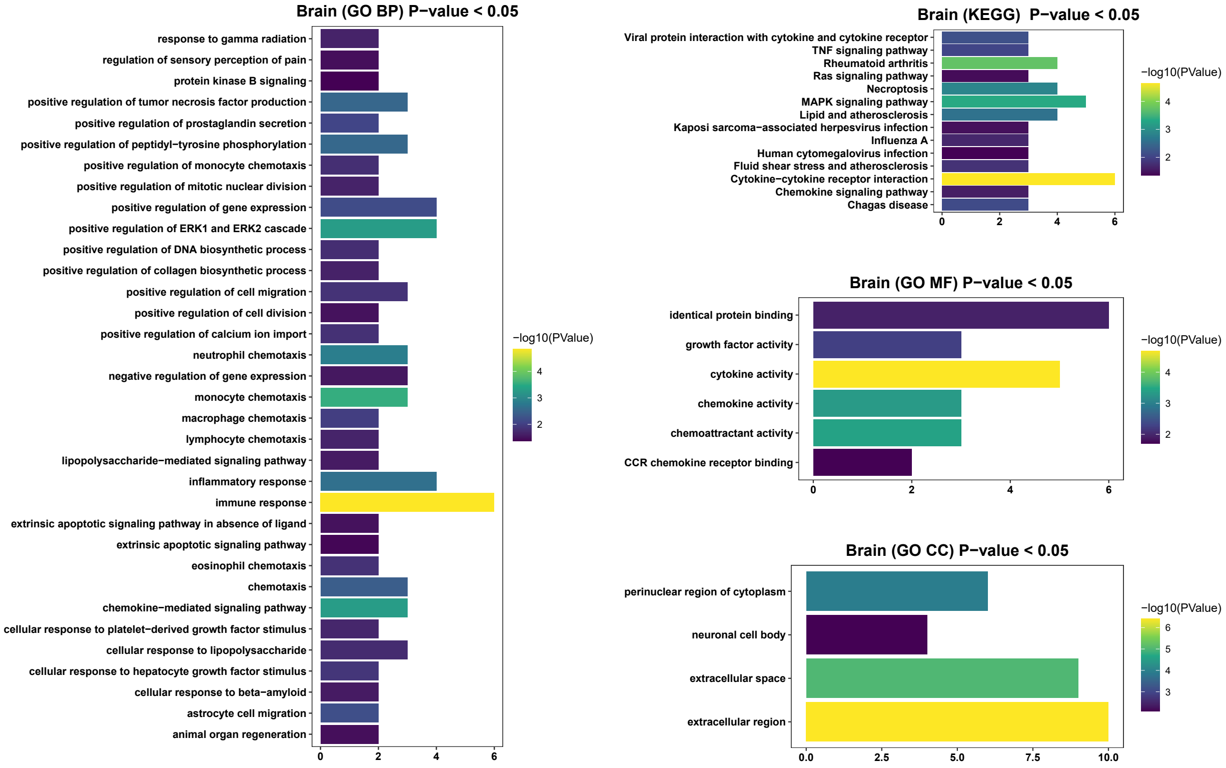


# DAVID Pathway Analysis - CSF

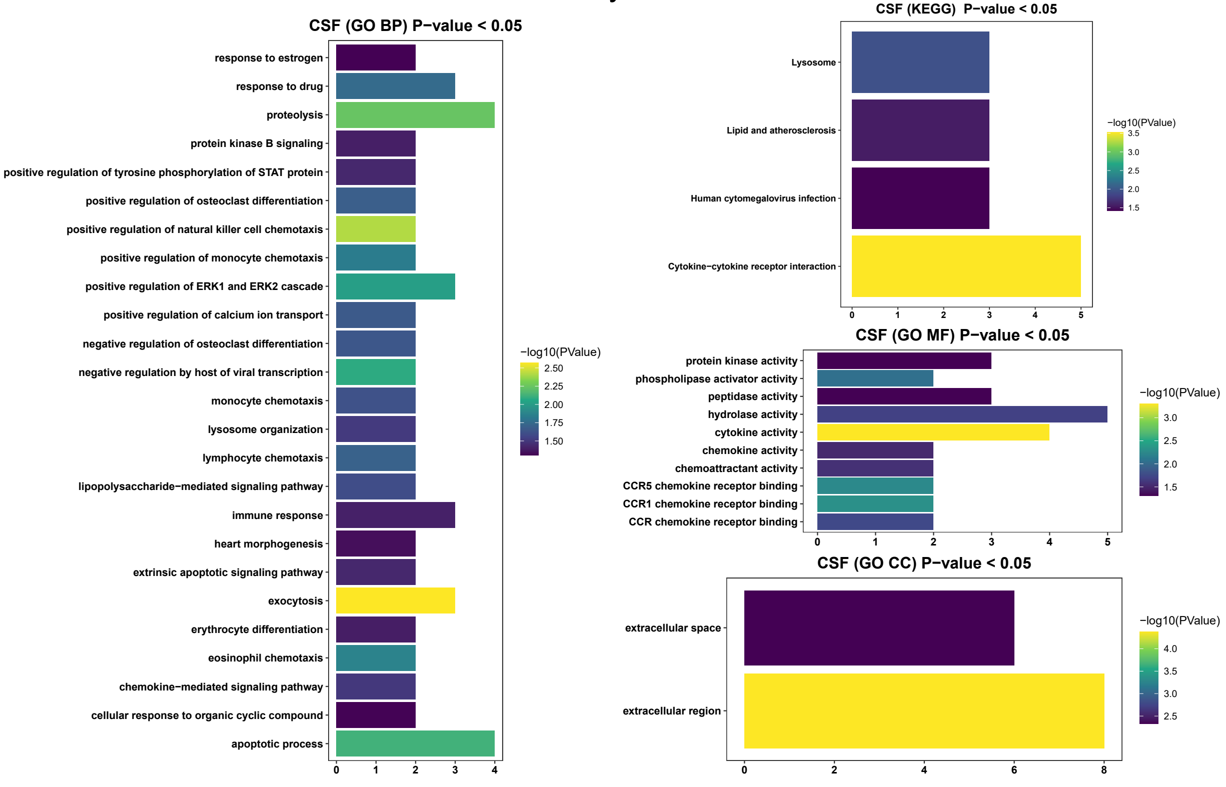

Supplement: Supplementary file 2 — Supplementary Figure S1. [file 41598_2023_42431_MOESM2_ESM.pdf]
