# Supplementary material for: Disease pathology signatures in a mouse model of Mucopolysaccharidosis type IIIB
Source: Sci Rep. 2023 Oct 4;13:16699. doi: 10.1038/s41598-023-42431-4 (PMC10550979; doi:10.1038/s41598-023-42431-4)
Supplement: Supplementary file 5 — Supplementary Legends. [file 41598_2023_42431_MOESM5_ESM.docx]

**SUPPLEMENTARY INFORMATION**

Additional file 1: **Supplementary Table S1_Complete dataset results from Olink proteomic analysis.xlsx**

Additional file 2: **Supplementary Figure S1_DAVID pathway results for Brain and CSF.pdf**

Additional file 3: **Supplementary Table S2_DAVID pathway results for Brain.xlsx**

Additional file 4: **Supplementary Table S3_DAVID pathway results for CSF.xlsx**
